# Evaluation of a zinc chelate on clinical swine dysentery under field conditions

**DOI:** 10.1186/s40813-019-0140-y

**Published:** 2020-01-16

**Authors:** Frédéric Vangroenweghe, Liesbeth Allais, Ellen Van Driessche, Robbert van Berkel, Gerwen Lammers, Olivier Thas

**Affiliations:** 1 Elanco, BU Food Animals, Plantijn en Moretuslei 1 – 3rd floor, 2018 Antwerpen, Belgium; 2 DGZ-Vlaanderen, Industrielaan 29, 8820 Torhout, Belgium; 3 Intracare BV, Voltaweg 4, 5466 AZ Veghel, The Netherlands; 4 I-BioStat, Data Science Institute, Hasselt University, Campus Diepenbeek, Agoralaan gebouw D, 3590 Diepenbeek, Belgium; 5 Department of Data Analysis and Mathematical Modelling, Faculty of Bioscience Engineering, Ghent University, Coupure Links 653, 9000 Ghent, Belgium; 6 National Institute of Applied Statistics Research Australia (NIASRA), University of Wollongong, Northfields Ave, Wollongong, NSW 2522 Australia

**Keywords:** Zinc chelate, *Brachyspira hyodysenteriae*, Swine dysentery

## Abstract

**Background:**

*Brachyspira hyodysenteriae* is the primary cause of swine dysentery, characterized by bloody to mucoid diarrhea due to mucohaemorhagic colitis in pigs and primarily affects pigs during the grow/finishing stage. Control and prevention of *B. hyodysenteriae* consists of administration of antimicrobial drugs, besides management and adapted feeding strategies. A worldwide re-emergence of the disease has recently been reported with an increasing number of isolates demonstrating decreased susceptibility to several crucially important antimicrobials in the control of swine dysentery. A novel non-antibiotic zinc chelate has been reported to demonstrate positive effects on fecal quality and consistency, general clinical signs, average daily weight gain and *B. hyodysenteriae* excretion during and after a 6-day oral treatment. The objective of the present study was to evaluate the zinc chelate (Intra Dysovinol^®^ 499 mg/ml (ID); Elanco) on naturally occurring swine dysentery due to *B. hyodysenteriae* under field conditions in the Netherlands.

**Results:**

Oral administration of zinc chelate resulted in improvement of general clinical signs from 3 days onwards in the ID-treated group combined with a significantly better total fecal score at 14 days post-treatment. Overall, average daily weight gain was better in the ID-treated group over the entire study period (0–14 days) and during the 8 days following the end of ID-treatment. A significant reduction (4.48 vs. 0.63 log_10_ cfu/g feces; ID-treated vs. control) in *B. hyodysenteriae* excretion was observed during the 6-day treatment period with a high percentage of animals (58.3 vs. 12.3%; ID-treated vs. control) with no excretion of *B. hyodysenteriae* from their feces. No additional antimicrobial treatment was needed in the ID-treated group, whereas 35% of the pigs in the control group were treated with an antibiotic at least once. No mortality occurred in both groups. No adverse events were reported during and following the ID-treatment.

**Conclusions:**

Zinc chelate – administered as a Zn-Na_2_-EDTA complex – is a non-antibiotic treatment for swine dysentery that reduces *B. hyodysenteriae* shedding with 4.48 log_10_ cfu/g feces within its 6-day treatment while improving general clinical signs (90.0 vs. 73.6% animals with normal score) and total fecal score within 2–4 days following administration in naturally infected pigs. The positive effects of ID treatment remain for at least 8 days after cessation of oral ID therapy. Pigs remaining in a highly contaminated environment may be re-infected following the end of ID treatment, however, this is not different to standard antimicrobial therapy. Therefore, control of swine dysentery should combine an efficacious treatment with additional management practices to reduce the environmental infection pressure in order to limit re-infection as much as possible. The ID treatment resulted in a higher growth rate and improved general health, whereas no mortality was observed and no additional therapeutic treatments were necessary in contrast to the control pigs.

## Background

*Brachyspira hyodysenteriae* (*B. hyodysenteriae*) – a β-haemolytic Gram-negative oxygen-tolerant anaerobic spirochete – is the primary cause of swine dysentery, characterized by bloody to mucoid diarrhea due to mucohaemorhagic colitis is pigs [1]. Swine dysentery primarily affects pigs during the growth and finishing period. Transmission of *B. hyodysenteriae* occurs through the fecal-oral route and is associated with several risk factors such as introduction of colonized animals (carriers), poor external (quarantine, rodents, wild birds and other potential reservoirs) and internal biosecurity measures (adequate cleaning and disinfection protocols, mixing of age groups) [2]. Clinical signs usually start with loss of appetite and mild, yellow to grey-coloured diarrhea, further progressing to watery diarrhea with blood, mucus and pseudomembranes [3]. This results in economic damage due to growth losses, mortality, increased variation in pig weight and decreased feed conversion at farm level.

Control and prevention of *B. hyodysenteriae* mainly consists of administration of antimicrobial drugs, besides management and adapted feeding strategies [4]. Currently, no commercial vaccines against *B. hyodysenteriae* are available [1], although some experimental vaccines, such as bacterins, subunit vaccines and live attenuated strains have been evaluated [5]. Recently, *B. hyodysenteriae* has been reported as a worldwide re-emerging disease with an increasing number of isolates having decreased susceptibility to several crucially important antibiotics in the control of swine dysentery [5–15].

Overall, control and prevention of *B. hyodysenteriae* seems to become more challenging, due to the limited treatment options [2], the lack of effective preventive feeding strategies and the increased awareness on reduction of antimicrobial use in animal production [16].

Consequently, research focused on non-antibiotic alternatives to reduce bacterial infections in general and *B. hyodysenteriae* infections in particular becomes more prominent. Adapted feeding strategies, including a high dietary concentration of inulin, have proven to reduce the incidence of swine dysentery due to *B. hyodysenteriae* in grower pigs [17]. A citrus extract commercialized as raw material and used as feed additive showed relevant in vitro bacteriostatic and bactericidal activity against *B. hyodysenteriae* at relatively low concentrations of 32 and 128 ppm, respectively [18]. Among others zinc has been evaluated as a potential intervention to control *B. hyodysenteriae*. In vitro addition of either ZnSO_4_ or CuSO_4_ to the growth medium of *B. hyodysenteriae* caused complete inhibition of hemolytic activity in 3 culture cycles. Further research revealed that the inhibition of hemolysin was specifically mediated by Zn^2+^ [19]. A comparative study with ZnSO_4_, Zn-methionine and ZnO only demonstrated a prophylactic effect of high concentrations of in-feed ZnO (2000 ppm or higher) against *B. hyodysenteriae* in a mouse challenge model for swine dysentery [20]. However, in 2017, the Committee for Medicinal Products of Veterinary Use concluded that the benefits of ZnO for the prevention of diarrhea in pigs do not outweigh the environmental risks of the product [21]. The recent withdrawal of the marketing authorization of ZnO by the European Commission limits the availability of effective alternatives to antimicrobial drugs. Consequently, there is a continuing need for new, effective, non-antibiotic innovations to further improve animal health and welfare and to help reducing economic losses due to *B. hyodysenteriae* infections in pigs.

Chelation of zinc with an organic molecule to form a Zn-Na_2_-EDTA – instead of covalent binding of zinc to inorganic oxygen – reduces its environmental impact [22]. In addition, previous in vitro (unpublished data) and in vivo studies [22] have demonstrated that the Zn-Na_2_-EDTA chelated complex – in relatively low concentrations – is potentially able to reduce adverse effects due to *B. hyodysenteriae* infections in pigs. An in vivo feasibility study demonstrated a positive effect of the zinc chelate to fecal quality and consistency, general clinical signs and average daily weight gain (ADWG) in *B. hyodysenteriae*-infected animals. Moreover, at the last treatment day, *B. hyodysenteriae* was not detectable by qPCR in most of the treated animals [22]. Based on these promising in vitro and in vivo results, the goal of the present study was to assess the efficacy of the zinc chelate, formulated as the Veterinary Medicinal Product Intra Dysovinol^®^ 499 mg/ml (ID; Elanco, Greenfield, IN) in the treatment of clinical signs due to *B. hyodysenteriae* infection in pigs under more challenging field conditions. For this purpose, the effect on excretion of *B. hyodysenteriae* was evaluated during and after a 6-day treatment period in two wean-to-slaughter units in the Netherlands.

## Materials and methods

### Inclusion of farms and animals

Two farms with clinical disease due to *B. hyodysenteriae* in grow/finisher pigs within two weeks preceding the start of the study or preventing clinical signs due to *B. hyodysenteriae* by strategic application of antimicrobial drugs (but showing relapse upon cessation of therapy) were included in this study. The clinical signs of swine dysentery in Farm 1 were mainly characterized by chronic diarrhea without very little presence of additions (mucus, necrotic material), occurrence of runt pigs and increased mortality. In Farm 2, the clinical signs of swine dysentery were more pronounced with bloody diarrhea, addition of mucus and necrotic material combined with retarded growth and finally mortality.

Pigs were not allowed to receive any preventive or curative antimicrobial drug for *B. hyodysenteriae* in the 10 days preceding Study Day (SD) 0 (first day of administration of ID). Farms were representative for Dutch commercial farms housing grow/finisher pigs under the highest welfare conditions (three stars within the welfare concept) with the possibility to realize administration of ID via drinking water using a dosing pump. Animals were fed dry feed and no increased levels of zinc or copper via the feed or drinking water were allowed. Pens included in the study had identical stocking density per pen, feed, climate and management.

A pen was included when at least 10% of the animals in the pen were qPCR-positive for *B. hyodysenteriae* at SD − 3 and at least one of the pigs was showing a non-normal fecal score (score 1 or higher on at least one aspect as described below). Only post-weaned pigs, showing non-normal fecal scoring and excreting *B. hyodysenteriae* at SD − 3 and/or at SD0 or SD2 (the latest) were included in the study for individual follow-up within selected pens.

### Administration of ID

Intra Dysovinol^®^ 499 mg/ml (Elanco) consists of 499 mg of Zn-Na_2_-EDTA per ml as an active ingredient in an aqueous solution further containing colorants and a preservative. Upon inclusion, the pen was randomly assigned to control or treatment with ID at a dosage of 0.023 ml product per kg bodyweight for the duration of 6 days (according to SPC specifications), starting at SD0 and ending at SD6 (Table 1). Based on the total bodyweight and total water consumption of all animals in a single pen, a 100 times concentrated pre-dilution was dosed at 1% to the drinking water using a calibrated dosing pump.

**Table 1:** Study outline indicating the actions performed on the different study days. I, individual level; P, pen level

		Study day									
Parameter	Level	-3	0	1	2	3	4	5	6	10	14
General health observations	P	X	X	X	X	X	X	X	X	X	X
Treatment	P		X	X	X	X	X	X	X		
Clinical observations	I		X	X	X	X	X	X	X	X	X
Faecal quality	I		X		X		X		X		X
qPCR faeces	I		X		X		X		X		X
Weighing	I		X						X		X

### Clinical observations and fecal quality

All animal observations and collection of animal samples were carried out by the observer. At pen level, general health observations on all pigs in the selected pens were recorded from SD − 3 until to SD14 (Table 1). Pigs were individually identified by unique ear tag numbers. All individually identified pigs in a pen were weighed on SD0, SD6 and SD14. Individual clinical observations were conducted at SD 0, 1, 2, 3, 4, 5, 6, 10 and 14 and scoring for general clinical condition, alertness, lameness and signs of respiratory diseases. For evaluation of the fecal quality, scoring was performed at SD 0, 2, 4, 6 and 14 according to the scoring grid in Table 2. Fecal scoring included consistency, color and additions (mucus, foam, blood and necrotic material) and was added to obtain the total fecal score (TFS).

**Table 2:** Fecal quality scoring grid for assessment of individual fecal quality. Sum (total fecal score, TFS) of fecal characteristics A, B and C was used for statistical analysis

Parameters	Score	Description
A. Faecal shape and consistency	1	Hard, dry, can be shaped by fingers
	2	Normal, soft, but not watery
	3	Pasty, soft
	4	Watery
B. Faecal color	0	Normal dark green, brown, feed color
	1	Light brown to yellow
	2	Grey
	3	Dark brown to black
C. Faecal additions	0	Normal, no additions
	1	Slimy or foamy
	2	Bloody or fibrinous exudate

### Real-time PCR and quantitative real-time PCR

Rectal fecal samples were collected at SD -3, 0, 2, 4, 6 and 14 in 50 ml sterile vials (MLS nv, Menen, Belgium). At SD − 3, the samples were analysed for the presence of B. hyodysenteriae by Real-Time PCR based on the nox gene. At all other sampling time points, the same real-time PCR was used combined with a standard curve in order to obtain a quantitative result (= quantitative real-time PCR). The cut-off Ct value is 37. For PCR analysis, DNA was extracted from 1 g of each individual faeces sample. Extraction was performed with the MagAttract 96 Cador Pathogen Kit (Indical Bioscience, Leipzig, Germany) according to the manufacturer’s instructions. Per g feces, 5 ml physiological solution was added to the sample, after which the mixture was thoroughly vortexed and 400 μl of supernatant was collected for extraction. The real-time PCR was performed using the BactoReal Kit *B. hyodysenteriae* (Ingenetix, Vienna, Austria), which was based on the *nox* gene of *B. hyodysenteriae*, using the ABI7500 detection system (Thermofisher, Massachusetts, USA) with the following cycling conditions: 2 min incubation at 50 °C and 20s incubation at 95 °C comprise the denaturation step, followed by 45 cycles of 95 °C for 15 s and 60 °C for 1 min. The fluorescence data were collected during the 60 °C – 1 min stage. The real-time PCR was based on TaqMan technology. A duplex PCR was performed using a probe labeled with the FAM™ dye for the target gene and a probe labeled with the Cy5® 186 dye for the internal positive control (IPC). This IPC was used to check for PCR inhibition. PCR detection limit was 18 copies of nucleic acid (i.e. 1.26 log_10_ cfu/g feces) per PCR reaction.

For quantification, the quantitative real-time PCR (qPCR) standard *B. hyodysenteriae* (Ingenetix, Vienna, Austria), containing 10^7^ copies/μl was used. Standard curves were performed using four dilution points: 10^6^–10^5^ – 10^4^ – 10^3^. Efficiency was between 85 and 115% and R^2^ was at least 0.99. Based on these standard curves, Ct values were translated into log_10_ cfu/gram feces (cfu = colony forming unit).

### Concomitant therapeutic treatment and unexpected death

If a pig enrolled in the study developed an abnormal health condition – such as profuse diarrhea, coughing or lameness – an individual therapeutic treatment was determined. In case an animal died or had to be euthanized because of severe illness/welfare reasons before SD0, the animal was disposed through regular channels without further investigation. In case of death/euthanasia after the treatment with ID or placebo had started, a fecal sample was collected for qPCR analysis (either immediately before euthanasia or as soon as possible after the pig was found dead) and examined post-mortem or disposed through regular channels.

### Justification of sample size

Sample size calculations for a two-sided two-sample t-test were carried out in JMP 14.1 using the following parameters based on a previous field study with ID in the Netherlands [22]:

- Observed standard deviation of 2 log_10_ cfu/g feces between both groups
- Calculations were performed using following statistical parameters:
  - Alpha 0.05
  - Power 95%
  - Minimal difference to be detected of 2 log_10_ cfu/g feces of *B. hyodysenteriae*

Based on these calculations, 60 pigs per treatment group was sufficient and therefore, 120 animals were enrolled in the entire study, equally distributed over 2 farms.

### Statistical analysis

The effect of treatment is tested for each time separately. To account for the variability between farms, the binary outcomes were analysed with a logistic regression model with treatment and farm as factors. The other outcomes were analysed with a linear regression model, again with treatment and farm as factors. All model parameters were estimated with the maximum likelihood method and the hypothesis tests were performed as Wald tests, which are for the linear regression model equivalent to the least squares method and t-test, respectively. The *P*-values (one for each time) were adjusted with the Bonferroni method so as to control the familywise error rate (FWER). All tests were performed at the nominal FWER level of 5%.

## Results

### Farms and animals

In total 122 pigs in 2 farms and 9 pens have been enrolled in the study for close, individual follow-up. Five of the initially selected pigs did not meet the inclusion criteria as they showed no excretion of *B. hyodysenteriae* at SD0. Therefore, data from 117 study pigs have been used to assess the efficacy of ID in the treatment of clinical signs due to *B. hyodysenteriae* infection under field conditions in the Netherlands. Of the 117 enrolled pigs, 60 received ID treatment (2 pens on farm A, 2 pens on farm B) and 57 received no treatment and were considered control (3 pens on farm A, 2 pens on farm B). On farm A, the individually followed pigs had an average bodyweight of 37.7 kg (range: 24.1–54.9 kg) on inclusion, while the pigs on farm B were younger with an average bodyweight of 15.2 kg (range: 9.4–22.4 kg). Bodyweights, distribution of sex (female/male), level of *B. hyodysenteriae* excretion and fecal quality score did not differ significantly at SD0 between ID-treated and control pigs as evaluated by the median test (*P* > 0.05) and the proportion test (*P* > 0.05), respectively.

### Clinical observations and fecal quality

**Fig. 1:**
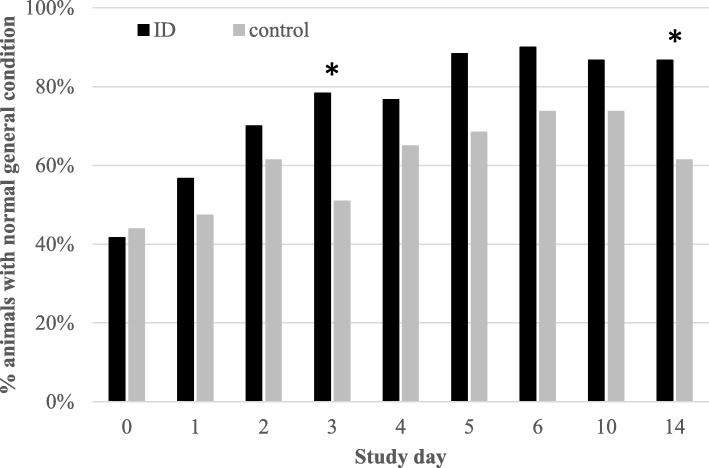
Percentage of ID-treated (*n* = 60) and control (*n* = 57) pigs with a normal general clinical score per study day from SD 0 to 14. Pigs were treated with ID from SD 0 to 6. Significant differences (*P* < 0.05) are indicated with asterix*

The general clinical score of the individually monitored pigs was comparable between ID-treated pigs and control pigs at SD0, with 25 animals in both group scoring normal (score 0). Following ID treatment, a rapid increase in the percentage of animals with a normal general clinical score occurred with 90% (54/60) of the animals scoring normal at SD6 (Fig. 1). In the control pigs, this general clinical score improved more slowly over time, resulting in 73.6% (42/57) of animals scoring normal at SD6. Following the end of ID treatment, the percentage of ID-treated pigs with a normal general clinical score remained relatively stable (~ 86.7%), while the percentage of control pigs with a normal general clinical score decreased significantly at SD14 (*P* < 0.05). The percentage of pigs with a normal general clinical score was significantly different (*P* < 0.05) between both groups from SD4 until SD14.

**Fig. 2:**
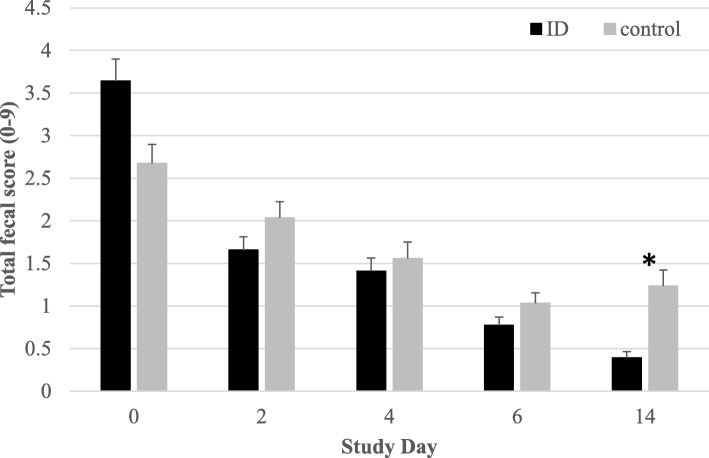
Total fecal score (average ± SEM) of ID-treated and control pigs per study day from SD 0 to 14. Pigs were treated with ID from SD 0 to 6. Significant differences (*P* < 0.05) are indicated with asterix*

The total fecal score (TFS) of the individually monitored pigs was comparable between ID-treated pigs and control pigs on SD0 with an average score of 3.16 and ranging from 0 to 7 for both treatment groups (t-test, *P* > 0.05). At SD0, TFS was numerically high in ID-treated pigs, however, from SD2 to SD6, ID-treated pigs had a lower TFS as compared to the control pigs (*P* > 0.05; Fig. 2). At SD14, 8 days after the end of the ID treatment, TFS in ID-treated animals (TFS 0.39) remained significantly lower (*P* < 0.01) as compared to control pigs (TFS 1.23), which was mainly due to differences in the scores of fecal consistency and fecal color. Overall, the ID-treated pigs showed an average daily improvement of their TFS by 0.74, whereas TFS in control pigs only had an average daily improvement of 0.38. Moreover, an increase in TFS was observed in control pigs at SD14. Overall, at SD6, 25 of the 60 ID-treated pigs showed a TFS of zero compared with 20 out of the 57 control pigs. At SD14, the number of ID-treated pigs with a TFS of zero increased to 39 out of 60, while in control pigs, this number remained at 20.

### Real-time semi-quantitative PCR

**Fig. 3:**
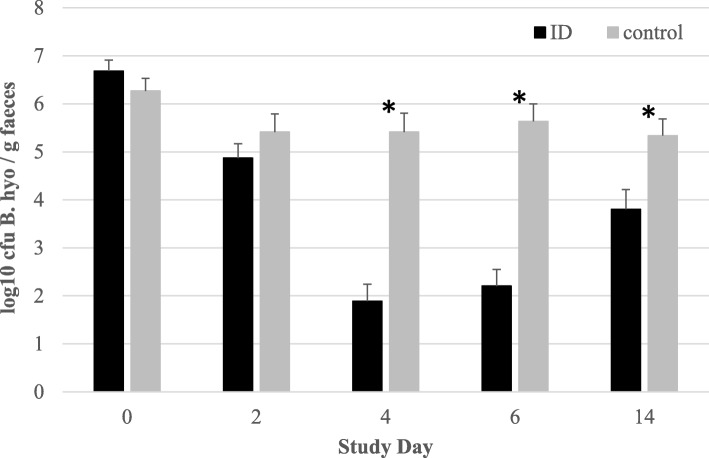
Log_10_ cfu *B. hyodysenteriae* per g of feces (average ± SEM) of ID-treated and control pigs per study day from SD 0 to 14. Pigs were treated with ID from SD 0 to 6. Significant differences (*P* < 0.05) are indicated with asterix*

**Fig. 4:**
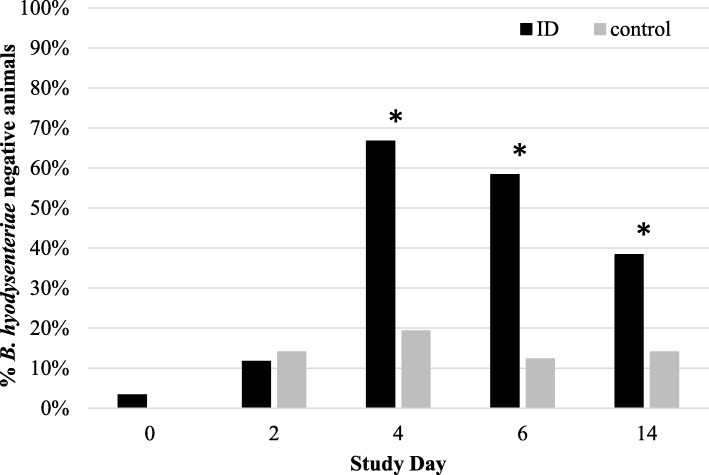
Percentage of *B. hyodysenteriae* PCR-negative ID-treated and control pigs per study day from SD 0 to 14. Pigs were treated with ID from SD 0 to 6. Significant differences (*P* < 0.05) are indicated with asterix*

*Brachyspira hyodysenteriae* nucleic acid was present at comparable levels in both treatment groups (6.68 log_10_ vs. 6.28 log_10_ cfu/g feces for ID-treated and control pigs, respectively) in all fecal samples collected at SD0, as determined by qPCR (t-test; *P* > 0.05, Fig. 3). At SD0, 2 out of 60 ID-treated pigs were *B. hyodysenteriae*-negative, while none of 57 control pigs were PCR-negative for the pathogen (Fig. 4). The number of *B. hyodysenteriae*-negative pigs in the ID-treated group remained equal to the control group at SD2, but increased to a maximum of 40 out of 60 at SD4. A slight fall-back in the number of PCR-negative ID-treated pigs (35/60) occurred at SD6, although their bacterial load (expressed as log_10_ cfu/g feces) decreased to 2.21 at SD6. Univariable analysis showed that *B. hyodysenteriae* nucleic acid excretion was significantly reduced in ID-treated pigs from SD4 to SD14 as compared to control pigs (t-test, *P* < 0.05). Multivariable analysis showed that the overall log_10_ cfu/g feces decreased significantly in ID-treated pigs from SD0 to SD6 compared to control pigs: ID-treated pigs showed a 4.48 log_10_ cfu/g feces reduction from SD0 to SD6, whereas control pigs only had a 0.63 log_10_ cfu/g feces reduction over the same period (*P* < 0.05).

### Average daily weight gain

**Fig. 5:**
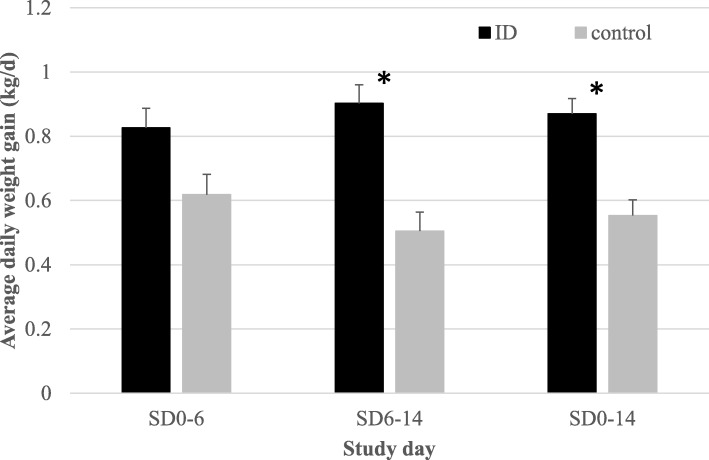
Daily weight gain (average ± SEM) of ID-treated and control pigs per study period. SD0–6, ID treatment; SD6–14, follow-up with ID treatment; SD0–14, entire study period. Significant differences (*P* < 0.05) are indicated with asterix*

The ID-treated pigs that were individually monitored had a significantly higher ADWG (ADWG, g/day) throughout the entire study as compared to the control pigs (Fig. 5). During the treatment period (SD0–6), ADWG in ID-treated pigs was at 825 g/day (± 61 g/d; SEM), while control pigs grew only 619 g/d (± 63 g/d; SEM). In the period following the end of ID treatment (SD6–14), ID-treated pigs increased to an ADWG of 903 g/d ((± 57 g/d; SEM), whereas ADWG in control pigs decreased to 505 g/day (± 59 g/d; SEM).

### Concomitant therapeutic treatment, unexpected death and adverse events

Due to persisting severe clinical symptoms of *B. hyodysenteriae* infection in 20 control pigs (35%), it was necessary to perform additional treatment with a single (*n* = 17) injection of tiamulin (Denagard 10%; Elanco) during the study. Three pigs needed more than one injection, namely two pigs were injected 3 times and one pig needed in total 4 injections of tiamulin. The ID-treated pigs did not require any additional treatment throughout the entire study and no mortality was observed in both treatment groups between SD0 and SD14. No adverse events related to ID treatment were observed during and after the study.

## Discussion

The current study demonstrated that treatment with zinc chelate (Intra Dysovinol^®^ 499 mg/ml; ID, Elanco) significantly reduced general clinical signs at SD3 and SD14, while shedding of *B. hyodysenteriae* was reduced with 4.48 log_10_ cfu per g feces during the treatment period, resulting in 58.3% *B. hyodysenteriae* negative animals on SD6. Treatment with ID for 6 consecutive days significantly improved TFS, which is an additive score of fecal consistency, color and eventual additions (mucus, foam, blood and necrotic material). The fecal quality remained good after the end of the 6-day ID treatment, although at SD14 an increase in *B. hyodysenteriae*-positive ID-treated pigs could be observed. This observation might be associated with the challenging housing circumstances of the pigs in the current study. In contrast to Lammers et al. (2019) [22], who performed his trials under conventional Dutch housing conditions, the pigs in our trial were housed in a concept including a high-welfare environment, which implicates more than 80% solid flooring, partly (50%) bedded with straw, and only a very little slatted surface. These circumstances do not favour the evacuation of *B. hyodysenteriae*-infected feces throughout the study and might expose the pigs to moderate to high amounts of potentially contagious fecal material. Moreover, a high percentage of pigs consumed liquid fecal material present on the solid pen floors.

In the current study, *B. hyodysenteriae* excretion did not completely disappear at SD6 in contrast to the results reported by Lammers et al. (2019) [22]. Besides the above mentioned factors related to differences in housing and environmental infection pressure, basic differences in sampling approach and subsequent qPCR analysis were present. First, the fecal samples in the current study were collected as a rather large volume of feces in a sterile vial, which allows the analytic laboratory to weigh 1 g of feces from each vial for DNA extraction and subsequent qPCR analysis. In contract, E-swabs were used by Lammers et al. (2019) [22], which implicates that potentially less than 1 g of fecal material was available for extraction. This difference in sampling approach might already influence the diagnostic sensitivity of the qPCR. Secondly, the PCR cut-off Ct value of 40 corresponded with a limit of detection and limit of quantification of 2.90 log_10_ cfu/g feces in the study by Lammers et al. (2019) [22], whereas in our study, the cut-off Ct value of 40 corresponded to 1.26 log_10_ cfu/g feces. Taken together, these differences in sampling and analysis might at least partially explain the observed difference in percentage of *B. hyodysenteriae*-negative animals between both studies at SD6.

The severity of the *B. hyodysenteriae* infection in both farms required additional veterinary intervention in the untreated control pigs. Before the start of the study, antimicrobial sensitivity to tiamulin was checked for the *B. hyodysenteriae* strains isolated and tiamulin MIC was at 0.25 μg/ml in both farms. Overall, 35% of the control pigs was administered one or more additional therapeutic treatments, whereas none of the ID-treated pigs required additional therapeutic intervention.

Although bodyweight of the animals at SD0 was different between farm A and B, no farm effect is expected on the results, since both treatments were equally distributed between both farms, resulting in similar bodyweights for both treatment groups. The fecal quality improved from 2 days after treatment onwards and continued TFS improvement was observed until 8 days after the end of ID treatment. This rapid improvement in clinical signs after ID treatment was in line with the 100-fold reduction in *B. hyodysenteriae* shedding within 2 days of ID treatment to an almost 10,000-fold reduction at 4 days in treatment. Although fecal scores continued to improved at SD6 and SD14, the qPCR results indicated a slight increase in *B. hyodysenteriae* shedding, which might be explained by the high environmental infection pressure due to the specific housing conditions. Considering the limit of detection by Lammers et al. (2019) [22], which was at 2.90 log_10_ cfu/g feces, our current qPCR results could also be considered ‘negative’ for *B. hyodysenteriae* shedding at the cut-off level of Ct value 40. Therefore, based on the clinical signs and fecal quality, we can conclude that the zinc chelate product had a sufficient efficacy in the treatment of swine dysentery due to *B. hyodysenteriae*.

The ability of *B. hyodysenteriae* to colonise the large intestine and its specific virulence factors are still not fully elucidated [2]. However, hemolysins, flagella, lipooligosaccharides and bacterial chemotaxis have been highlighted within the pathogenesis of swine dysentery, besides specific virulence life-style factors, such as outer membrane proteins, NADH oxidase and proteins of iron metabolism [1]. It requires further investigation which mechanism causes the novel zinc chelate to apparently prevent colonization and subsequently enhance the elimination of the pathogen [22]. In a mouse model for swine dysentery, the effect of zinc methionine, ZnO and ZnSO_4_ has been assessed and only ZnO levels of at least 2000 ppm demonstrated a prophylactic effect against *B. hyodysenteriae*, which is a considerably higher dose than what was required for the zinc chelate in the current study [20]. Another study reported no therapeutic effect of 250 ppm zinc chelate in the drinking water for 17 days to pigs inoculated with *B. hyodysenteriae*, which may be due to the nature of the chelating agent used [23].

Besides its impact on animal health and welfare, swine dysentery due to *B. hyodysenteriae* has a tremendous impact on the economic impact of an affected farm due to reduced pig performance, increased antimicrobial treatment costs and mortality. Annual losses of about € 133 per sow were calculated for fattening pigs affected by clinical swine dysentery [24]. In the current study, growth results of the control pigs were significantly affected by swine dysentery, although we could not observe weight losses, in contrast to the study by Lammers et al. (2019) [22]. The ID treatment had a significant positive impact on pig performances with an overall ADWG of 869 g/d from SD0 to SD14, while in the control pigs, the ADWG was only 553 g/d. These results indicate that the intestinal recovery at the level of the colon following ID treatment had a continued effect for at least 8 days after the end of ID treatment, which was clinically confirmed by the stable fecal quality and overall healthier appearance of the pigs in the ID-treated group.

Water medication is a convenient and flexible route of administration, permitting the farmer to apply the necessary treatment to a specific category of animals, resulting in an overall reduced use of therapeutics at farm level. Moreover, during a disease outbreak, water consumption remains stable for a much longer period as compared to feed intake, which implicates that disease animals can more efficiently be treated through this route of administration. During the trial, daily monitored water intake indicated that sick pigs continued to drink, while feed intake might be affected during the acute phase of *B. hyodysenteriae* infection [3].

## Conclusions

Intra Dysovinol^®^ 499 mg/ml – containing a Zn-Na_2_-EDTA complex – is a non-antibiotic treatment for swine dysentery due to *B. hyodysenteriae* that reduces *B. hyodysenteriae* shedding with 4.48 log_10_ cfu per g feces within its 6-day treatment. Treatment improved general clinical signs (90.0 vs. 73.6% animals with normal score in ID-treated vs. control) at SD6 and TFS (0.39 vs. 1.23 in ID-treated vs. control) at SD14 in naturally *B. hyodysenteriae* infected pigs. The positive effects of ID treatment remain for at least 8 days after cessation of oral ID therapy. Pigs remaining in a highly contaminated environment may be re-infected following the end of ID treatment, however, this is not different to standard antimicrobial therapy. Therefore, control of swine dysentery should combine an efficacious treatment with additional management practices to reduce the environmental infection pressure in order to limit re-infection as much as possible. The ID treatment resulted in a higher growth rate and improved general health, whereas no mortality was observed and no additional therapeutic treatments were necessary in contrast to the control pigs.

## Data Availability

The datasets analysed during the current study are available from the corresponding author on reasonable request.

## Abbreviations

ADWG: Average daily weight gain
*B. hyodysenteriae*: *Brachyspira hyodysenteriae*
cfu: Colony-forming unit
ID: Intra Dysovinol^®^
MIC: Minimal inhibitory concentration
qPCR: Quantitative polymerase chain reaction
SD: Study Day
SPC: Specified product characteristics
TFS: Total fecal score
